# A novel immune checkpoint score system for prognostic evaluation in pancreatic adenocarcinoma

**DOI:** 10.1186/s12876-023-02748-w

**Published:** 2023-04-06

**Authors:** Yusheng Chen, Xuan Lin, Xuan Zou, Yunzhen Qian, Yu Liu, Ruijie Wang, Xu Wang, Xianjun Yu, Chen Liu, He Cheng

**Affiliations:** 1 Department of Pancreatic Surgery, Shanghai Cancer Center, Fudan University, 270 DongAn Road, Xuhui, Shanghai 200032 People’s Republic of China; 2 Department of Oncology, Shanghai Medical College, Fudan University, Shanghai, 200032 China; 3 Shanghai Pancreatic Cancer Institute, Shanghai, 200032 China; 4 Pancreatic Cancer Institute, Fudan University, Shanghai, 200032 China

**Keywords:** Pancreatic adenocarcinoma, Immune checkpoints, Prognostic evaluation, OX40, Immune risk score model

## Abstract

**Background:**

Pancreatic adenocarcinoma (PAAD) remains a lethal malignancy making the detection of novel prognostic biomarkers urgent. Limited studies have investigated the predictive capability of immune checkpoints in PAAD.

**Method:**

Gene expression data and correlative clinical information of PAAD cohort were obtained from public databases, including TCGA, ICGC, GTEX and GEO databases. Risk factors were screened and used to establish a risk score model through LASSO and Cox regression analyses. The prognostic ability of the risk score model was demonstrated. The association between risk score with immune cells infiltration, immune checkpoint genes expression, immunogenic cell death, somatic mutations and signaling pathways enrichment were analysed. scRNA-seq data were collected to confirmed the immune checkpoints expression in PAAD samples. The prognosis prediction ability of OX40/TNFRSF4 was identified. The mRNA and protein expression of OX40 in our clinical specimens were examined by RT-PCR and IHC method and its prognosis ability was verified.

**Results:**

First of all, the difference of immune microenvironment between pancreatic cancer and adjacent tissues was shown. A risk score system based on three immune checkpoints (*OX40*, *TNFSF14* and *KIR3DL1*) was established. The risk score model was an independent prognostic factor and performed well regarding overall survival (OS) predictions among PAAD patients. A nomogram was established to facilitate the risk model application in clinical prognosis. Immune cells including naive B cells, CD8^+^ T cells and Tregs were negatively correlated with the risk score. The risk score was associated with expression of immune checkpoint genes, immunogenic cell death related genes and somatic mutations. Glycolysis processes, IL-2-STAT5, IL-6-STAT3, and mTORC1 signaling pathways were enriched in the high-risk score group. Furthermore, scRNA-seq data confirmed that *TNFRSF4*, *TNFSF14* and *KIR3DL1* were expressed on immune cells in PAAD samples. We then identified *OX40* as an independent prognosis-related gene, and a higher *OX40* expression was associated with increased survival rate and immune environment change. In 84 PAAD clinical specimens collected from our center, we confirmed that higher *OX40* mRNA expression levels were related to a good prognosis. The protein expression of OX40 on tumor-infiltrating immune cells (TIICs), endothelial cells and tumor cells was verified in PAAD tissues by immunohistochemistry (IHC) method.

**Conclusions:**

Overall, our findings strongly suggested that the three-immune checkpoints score system might be useful in the prognosis and design of personalized treatments for PAAD patients. Finally, we identified OX40 as an independent potential biomarker for PAAD prognosis prediction.

**Supplementary Information:**

The online version contains supplementary material available at 10.1186/s12876-023-02748-w.

## Introduction

Pancreatic ductal adenocarcinoma (PAAD) remains a highly aggressive malignancy with rapid progression and a high mortality rate [1, 2]. For example, the overall 5-year survival of PAAD patients is about 3–7% in the USA. In recent years, the incidence and mortality rates have also increased in China [3]. Besides, advances over the past decade in PAAD treatment strategies (diagnostic approaches, radiotherapy techniques, perioperative management) have made minor to modest improvements in patient outcomes [4]. Therefore, new strategies for high-risk PAAD patients screening are required to promote clinically significant impacts [5].

Increasing evidence indicated that the tumor immune microenvironment participates in cancer occurrence and progression [6]. Moreover, dysregulation of the immune system is involved in cancer cells escaping immune system recognition and elimination [7]. Checkpoint inhibitor-based immunotherapy has achieved impressive success in several cancer types. Unfortunately, the complex interactions between tumor cells, the desmoplastic stroma, and immune cells in pancreatic cancer create a highly immunosuppressive tumor microenvironment, finally resulting in insusceptibility to single-agent immune checkpoint blockades [8]. Increasing evidence has indicated an association between immune-related genes and clinical outcomes across different cancer types such as lung, colorectal, and ovarian cancers [9–11]. These studies showed that immune-related genes can be promising biomarkers for tumor prognosis. However, it is still unknown whether immune checkpoints are useful in predicting PAAD prognosis. Thus, it is meaningful to use valuable immune checkpoints to build a risk scoring system to evaluate the risk and prognosis of PAAD patients.

In the present study, we developed a new risk score model for PAAD with predictive power based on immune checkpoints via bioinformatics analyses. First, RNA-Seq data from the Cancer Genome Atlas (TCGA) and Gene Expression Omnibus (GEO) databases were retrieved and analyzed. The least absolute shrinkage and selection operator (LASSO) method was used to create a three-immune checkpoint (OX40, TNFSF14, and KIR3DL1) risk scoring system for PAAD. Then, the relationship between the model and overall survival (OS) was investigated. A nomogram was established for survival predictions. Immune cell composition and signaling pathways were also analyzed between different risk groups. Single-cell datasets(TISCH and CancerSCEM) confirmed that *TNFRSF4*, *TNFSF14* and *KIR3DL1* were expressed on PAAD-related immune cells. Furthurmore, we identified OX40 as an independent prognostic biomarker, which was further validated in our 84 clinical specimens. Overall, our current results might provide a new reference for PAAD prognosis prediction.

## Results

### Infiltration of immune cells and expression of immune checkpoint genes in PAAD

**Fig. 1:**
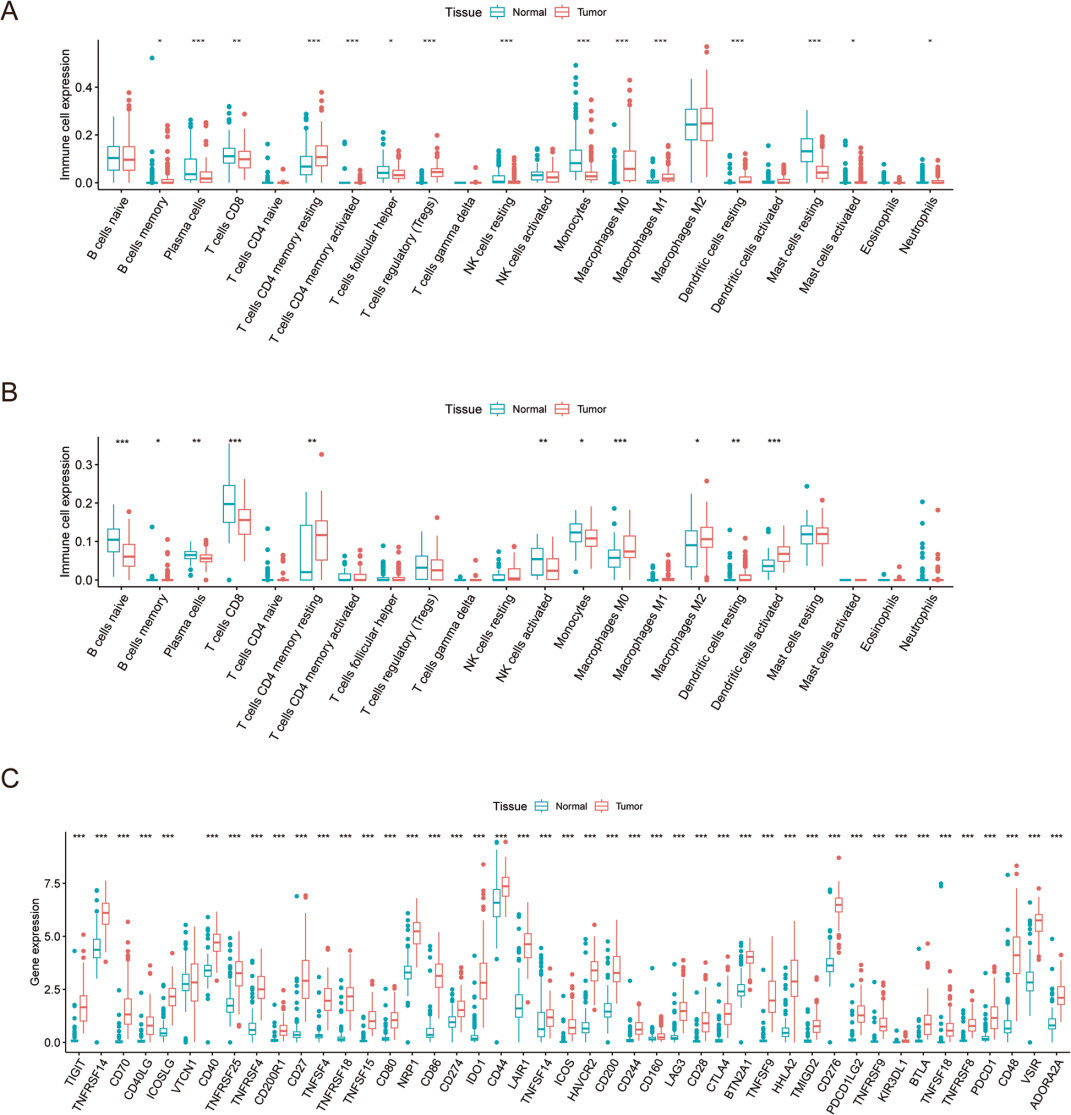
Expression of tumor-infiltrating immune cells and immune checkpoint genes between PAAD and normal cases. **A** Differences in the abundance of tumor-infiltrating immune cells between PAAD and normal tissues calculated by CIBERSORT in TCGA and GTEX database. **B** Differences in the abundance of tumor-infiltrating immune cells between PAAD and normal tissues calculated by CIBERSORT in GSE62452 dataset. **C** Differences in the expression of immune checkpoint genes between PAAD and normal tissues. *p < 0.05, **p < 0.01, ***p < 0.001

First, we retrieved RNA-sequencing (RNA-seq) expression data and relevant clinical information for 183 PAAD samples from the TCGA database, including 179 tumor cases and 4 normal cases. In addition, 167 normal pancreatic tissue were obtained from GTEX database. According to CIBERSORT algorithm, 123 samples were screened and included. The clinicopathological characteristics of included patients are summarized in Table 1. The original tumor type table was shown in Additional file 1. To explore the immune microenvironment role, we evaluated the composition of TIICs in PAAD samples (Fig. 1). In the TCGA database, there were 11 kinds of immune cells differentially infiltrating between pancreatic cancer and adjacent tissues according to the CIBERSORT algorithm (Fig. 1A).In GSE62452 dataset, a total of 5 immune cells(memory B cells, memory resting CD4 T cells, M0 macrophages, M2 macrophages, activated dentritic cells) were highly infiltrated in pancreatic cancer tissues, while 6 kinds of immune cells are highly infiltrated in normal tissues (naïve B cells, plasma cells, CD8 T cells, activated NK cells, monocytes, resting dentritic cells) (Fig. 1B). Next, we analyzed the expression of immune checkpoint-related genes. We found significant differences in immune checkpoints expressions between PAAD and normal tissues (Fig. 1C). The above findings indicated that compared with normal pancreatic tissue, pancreatic cancer tissue had a unique immune microenvironment, suggesting that immune related events played an important role in pancreatic cancer.

**Table 1:** Clinical characteristics of included patients in the study

Variables	Total (*n* = 123)	Training cohort (*n* = 63)	Validation cohort (*n* = 60)
Status			
Dead	62(50.41%)	29(46.03%)	33(55%)
Alive	61(49.59%)	34(53.97%)	27(45%)
Age			
< 65	53(43.09%)	27(42.86%)	26(43.33%)
> = 65	70(56.91%)	36(57.14%)	34(56.67%)
Gender			
FEMALE	59(47.97%)	29(46.03%)	30(50%)
MALE	64(52.03%)	34(53.97%)	30(50%)
Stage			
I	12(9.76%)	8(12.7%)	4(6.67%)
II	104(84.55%)	52(82.54%)	52(86.67%)
III	3(2.44%)	2(3.17%)	1(1.67%)
IV	3(2.44%)	1(1.59%)	2(3.33%)
Unknown	1(0.81%)	0(0%)	1(1.67%)
Grade			
G1	14(11.38%)	7(11.11%)	7(11.67%)
G2	69(56.1%)	36(57.14%)	33(55%)
G3	39(31.71%)	19(30.16%)	20(33.33%)
G4	1(0.81%)	1(1.59%)	0(0%)
T			
T1	4(3.25%)	3(4.76%)	1(1.67%)
T2	17(13.82%)	10(15.87%)	7(11.67%)
T3	99(80.49%)	48(76.19%)	51(85%)
T4	3(2.44%)	2(3.17%)	1(1.67%)
M			
M0	66(53.66%)	32(50.79%)	34(56.67%)
M1	3(2.44%)	1(1.59%)	2(3.33%)
MX	54(43.9%)	30(47.62%)	24(40%)
N			
N0	31(25.2%)	17(26.98%)	14(23.33%)
N1	92(74.8%)	46(73.02%)	46(76.67%)

### Construction of an Immune checkpoint-based prognostic model based on LASSO and Cox analyses

**Fig. 2:**
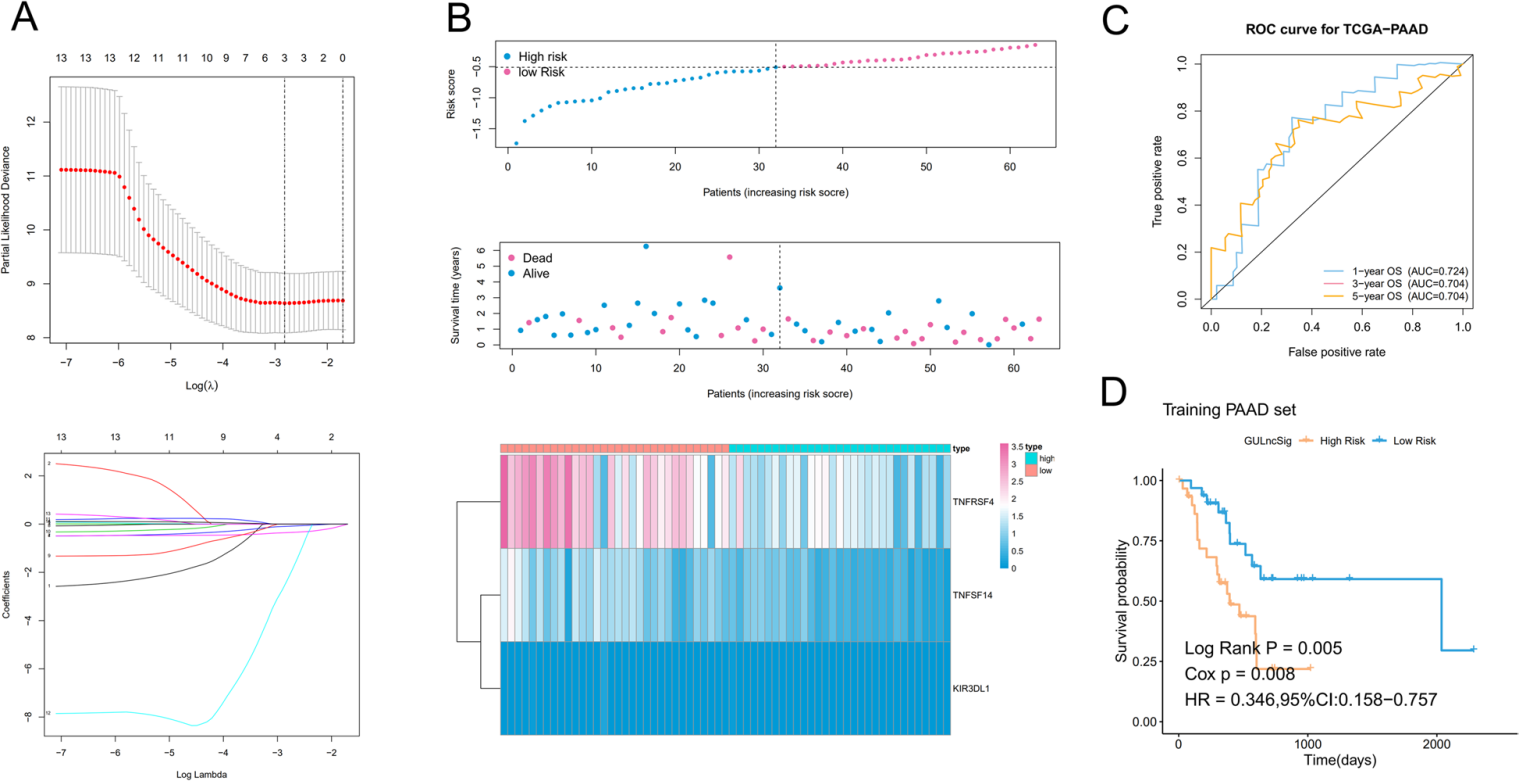
Three immune checkpoint genes were screened to construct the immune risk scoring model. **A** The LASSO regression analysis identified three immune checkpoint genes with the best prognostic value to be incorporated into the prognostic signature. **B** Distribution of immune risk scores, survival status, and heatmap of gene expression patterns (*TNFRSF4*, *TNFSF14*, and *KIR3DL1*) in the high-risk and low-risk groups in the training cohort. **C** ROC curve in the training cohort. **D** Kaplan–Meier survival curves of overall survival in the training cohort

Foremost, we randomly divided patients into a training set and a testing set. Three genes (*TNFRSF4*, *TNFSF14*, and *KIR3DL1*) were selected using LASSO regression analysis from the PAAD cohort based on the abovementioned immune checkpoint-related genes and incorporated into the prognostic gene signature. The risk score formula was: risk score = TNFRSF4 expression X (-0.0913) + *TNFSF14* expression X (-0.353) + *KIR3DL1* expression X (-2.317) (Fig. 2A). According to the median value of the prognostic risk score, the training cohort was classified into high and low-risk groups. The distribution of immune risk score, survival status of each subject, as well as a heatmap of the gene expression pattern, are presented in Fig. 2B. The ROC curve indicated that the 1-year AUC was 0.724, the 3-years AUC was 0.704, and the 5-years AUC was 0.704. These results demonstrated that the risk model can be used for PAAD survival prediction with good accuracy (Fig. 2C). The Kaplan–Meier survival analysis of the training cohort showed that patients in the low-risk group had significantly better OS than those in the high-risk group (*p* = 0.005) (Fig. 2D).

**Fig. 3:**
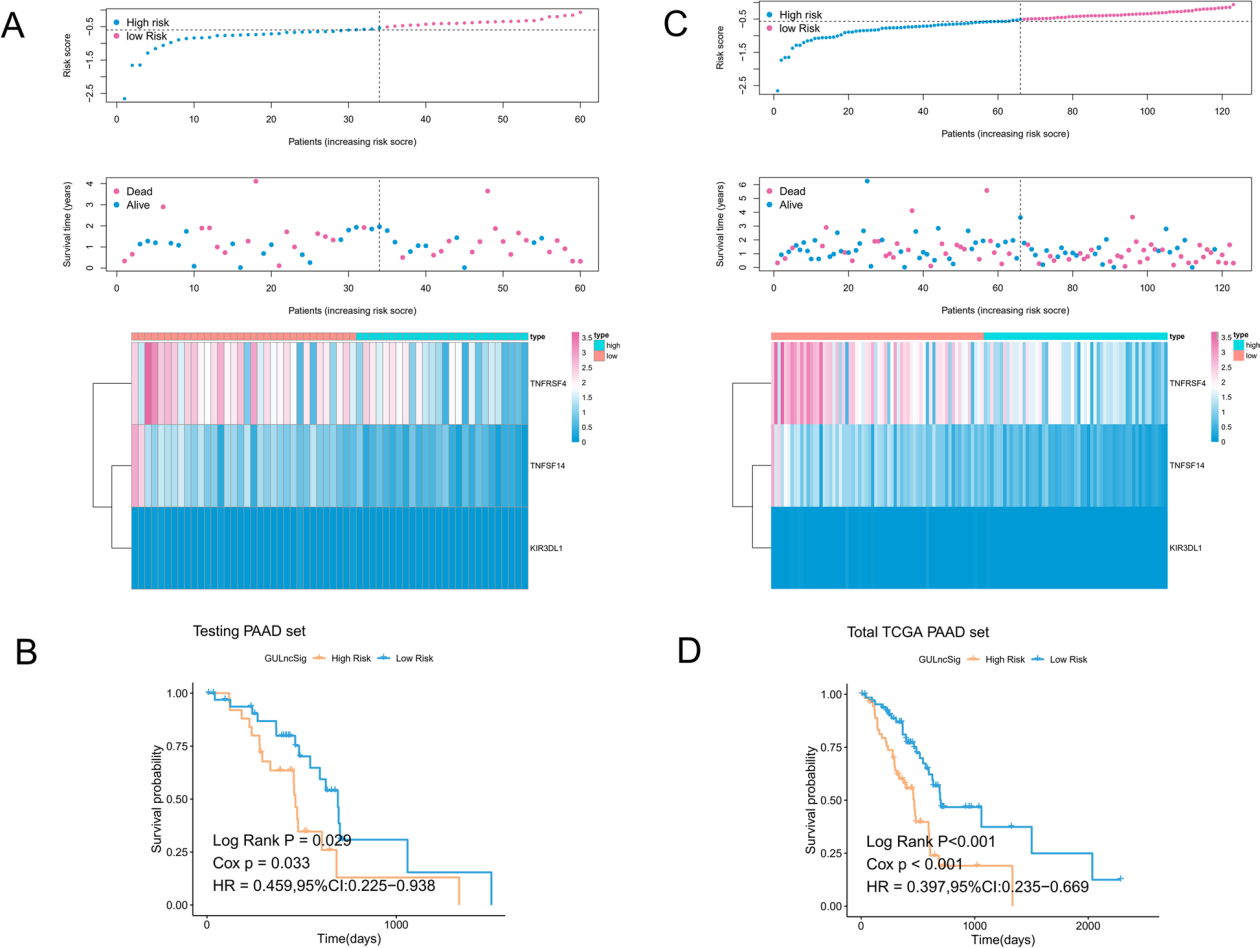
Validation of the risk scoring model in testing and total cohorts. **A** Risk score distribution, survival time, and expression patterns of the three genes in risk groups in the testing cohort. **B** Kaplan–Meier survival curves between different risk groups in the testing cohort. **C** Distribution of risk score, survival time, and heatmap of the expression of the three genes in the total cohort. **D** Kaplan–Meier survival curves in the total cohort

Then, based on the same risk score formula and cut-off value, the predictive capability of the immune checkpoint-related risk model was verified in the test cohort. The risk score, survival time, and gene expression value in the testing cohort are presented in Fig. 3A. The Kaplan–Meier plot of the testing cohort confirmed that patients in the high-risk group had a significantly poorer OS(*p* < 0.05) (Fig. 3B).

Besides, the robustness of this three-immune checkpoint risk score model was assessed in the total cohort. The prognostic predictions for patients in the total cohort presented similar results. The distribution of risk score, survival status, and expression of the three immune-related genes in the total cohort are shown in Fig. 3C. Moreover, high-risk patients had a shorter median survival time than low-risk ones (*p* < 0.001) (Fig. 3D).

### The immune checkpoint-based risk scoring model was an independent prognostic factor for PAAD

**Fig. 4:**
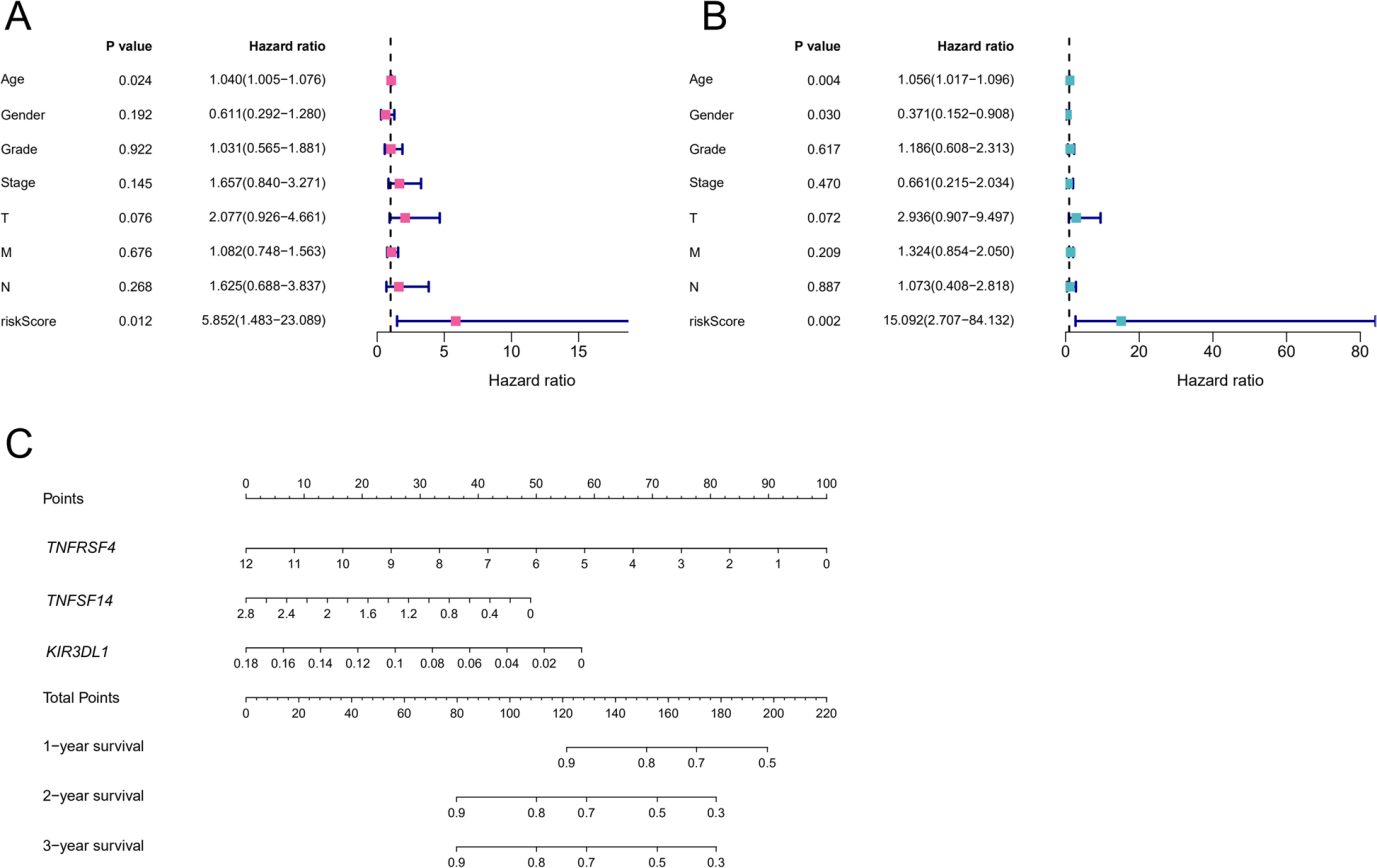
Cox’s model of related factors in PAAD patients. **A** Univariate Cox analysis for eight OS-related clinicopathological parameters. **B** Multivariate Cox analysis for eight OS-related clinicopathological parameters. **C** A prognostic nomogram predicting the OS of PAAD patients. OS: overall survival

To investigate whether the immune checkpoint-based prognostic model was independently associated with OS, we conducted Cox regression analyses including as variates patients’ age, gender, tumor grade, stage, TNM, and risk score (Fig. 4). The univariate Cox analysis showed that age and high immune risk score were related to poor OS (Fig. 4A). The multivariate Cox analysis results revealed that several factors (age, gender, and immune checkpoint-based risk score) were independently associated with prognosis (Fig. 4B). Then, a comprehensive prognostic nomogram was conducted to predict the survival of PAAD patients based on the multivariate Cox analysis coefficients (Fig. 4C).

### The immune risk score was associated with tumor immune microenvironment changes

**Fig. 5:**
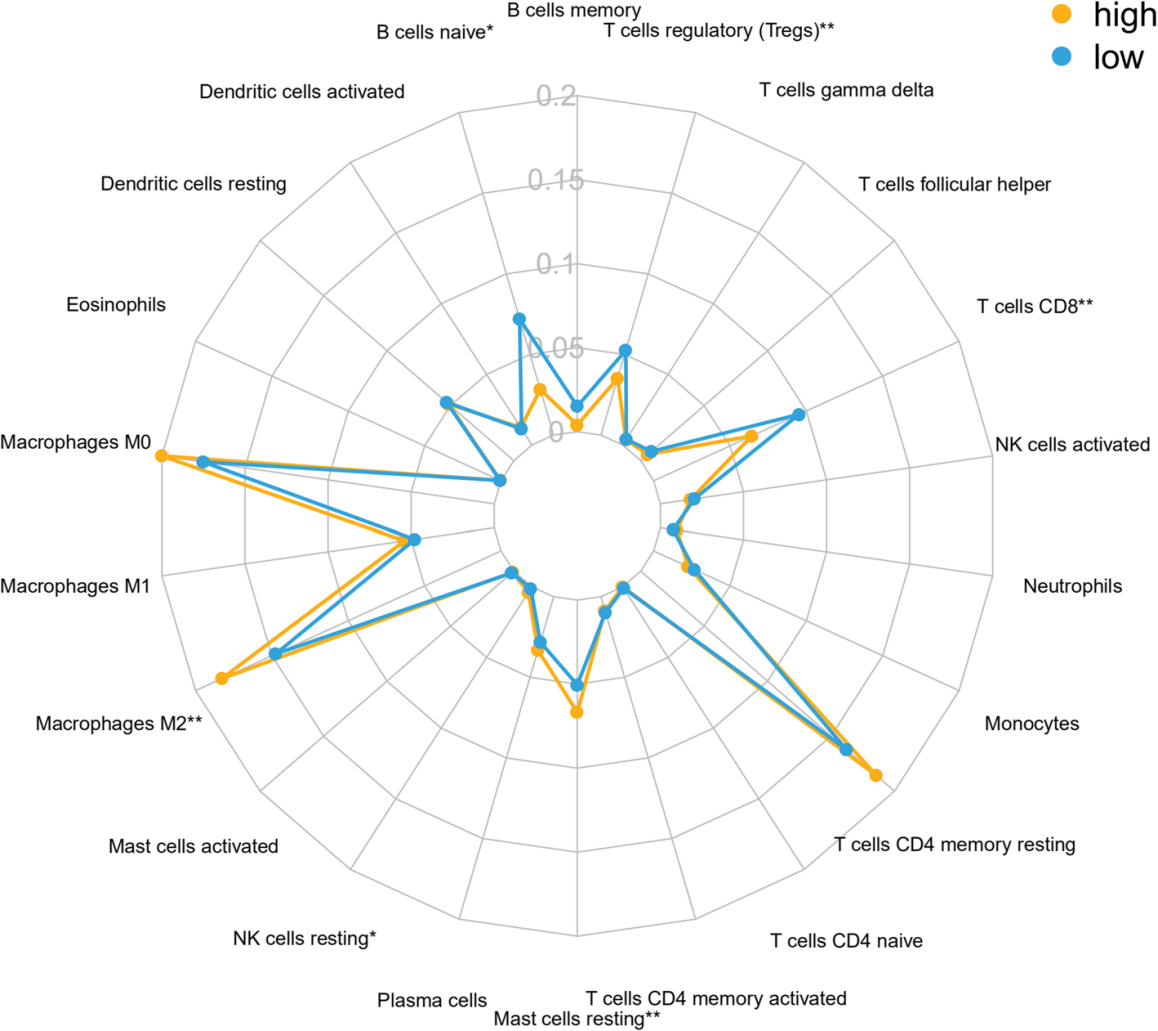
Relationship between immune checkpoint-related risk scores and immune cells infiltration in PAAD. Radar graph of the relative abundance of immune cells in different risk groups in PAAD samples

**Fig. 6:**
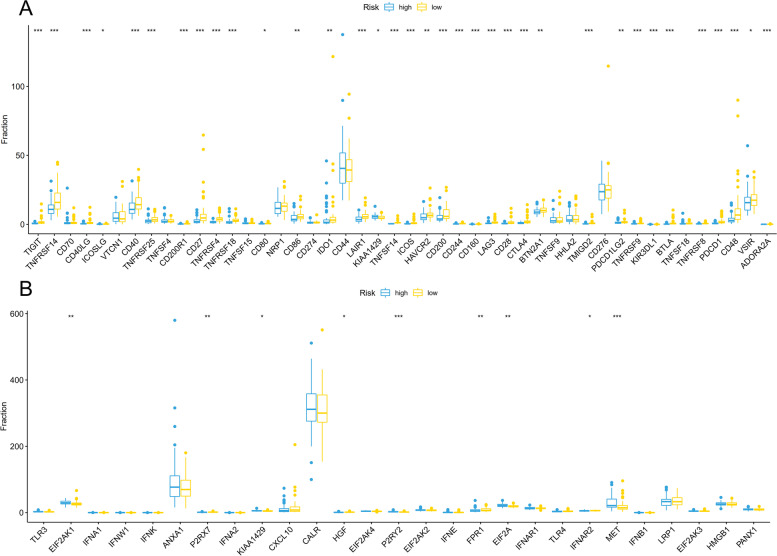
Relationship between risk scores with immune checkpoint and immunogenic cell death-related gene expression in PAAD. **A** Differentially expressed immune checkpoint-related genes in different risk groups. **B** Differentially expressed immunogenic cell death-related genes in different risk groups

Immune cells are the major component of the tumor immune microenvironment. Hence, we evaluated if this immune risk score system was associated with immune cell infiltration changes. CIBERSORT was applied to assess the infiltration of immune cells in each PAAD sample. The abundance of each immune cell type between high-risk and low-risk groups in the total cohort is presented in Fig. 5. Among the 22 immune cell types, naive B cells, CD8 T cells, and Tregs were negatively correlated with the immune-related risk score. Additionally, M2 macrophages, resting NK cells, and resting mast cells showed a positive association with the risk score (Fig. 5). Moreover, studies have demonstrated the key role of immunogenic cell death (ICD) in the pancreatic tumor microenvironment in stimulating antitumor immune responses. The differentially expressed immune checkpoint and ICD-related genes between high and low-risk groups are presented (Fig. 6). These results might account for the poorer prognosis of PAAD patients in the high-risk group.

### Gene set enrichment analysis (GSEA) and Mutation profile

**Fig. 7:**
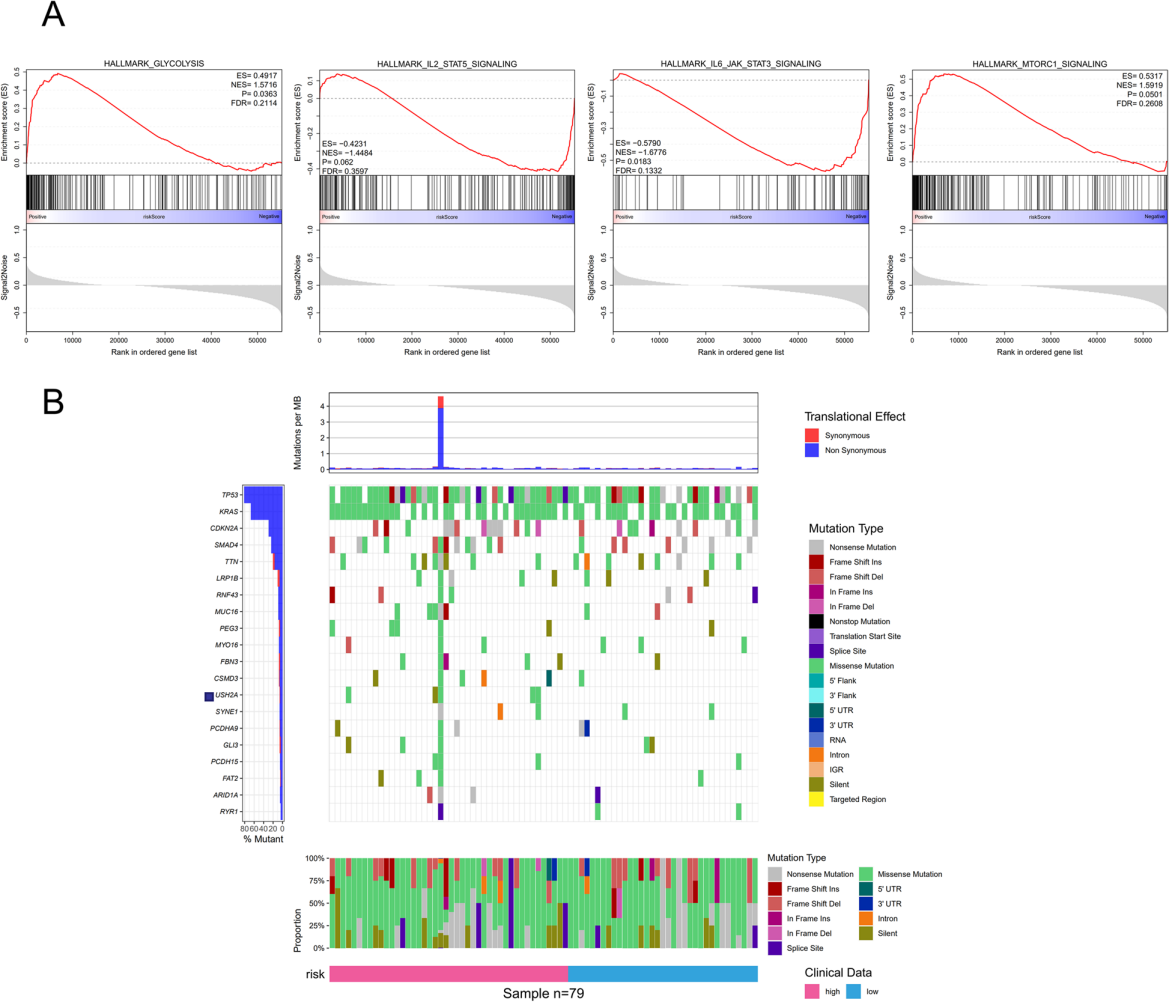
GSEA enrichment plots and mutation profile between different risk groups. **A** The GSEA was based on the risk score median value in PAAD patients. **B** The gene mutation frequency is shown on the left panel, while the mutational prevalence of synonymous/nonsynonymous mutations is presented on the upper panel. The gene mutation landscape is displayed in the middle. Different risk groups are depicted on the bottom

To analyze the mechanisms underlying different immune checkpoint-related risk scores, we conducted a GSEA between different risk score groups to identify potential immune-related biological functions and pathways. Results revealed that in the high-risk score group glycolysis, IL-2-STAT5, IL-6-STAT3, and the mTORC1 signaling were enriched (Fig. 7A). Their corresponding biologic functions might contribute to PAAD patients’ different risks and prognoses. Next, we explored the relationship between mutation profile and risk score with available somatic mutation data. The top 10 mutated genes in PAAD patients were: *TP53, KRAS, CDKN2A, SMAD4, TTN, LRP1B, RNF43, MUC16, PEG3,* and *MYO16*. The most frequently mutated genes in both risk groups are presented in Fig. 7B. Mutations in *USH2A* were only found in the high-risk group.

### Single-cell mRNA-sequencing data

**Fig. 8:**
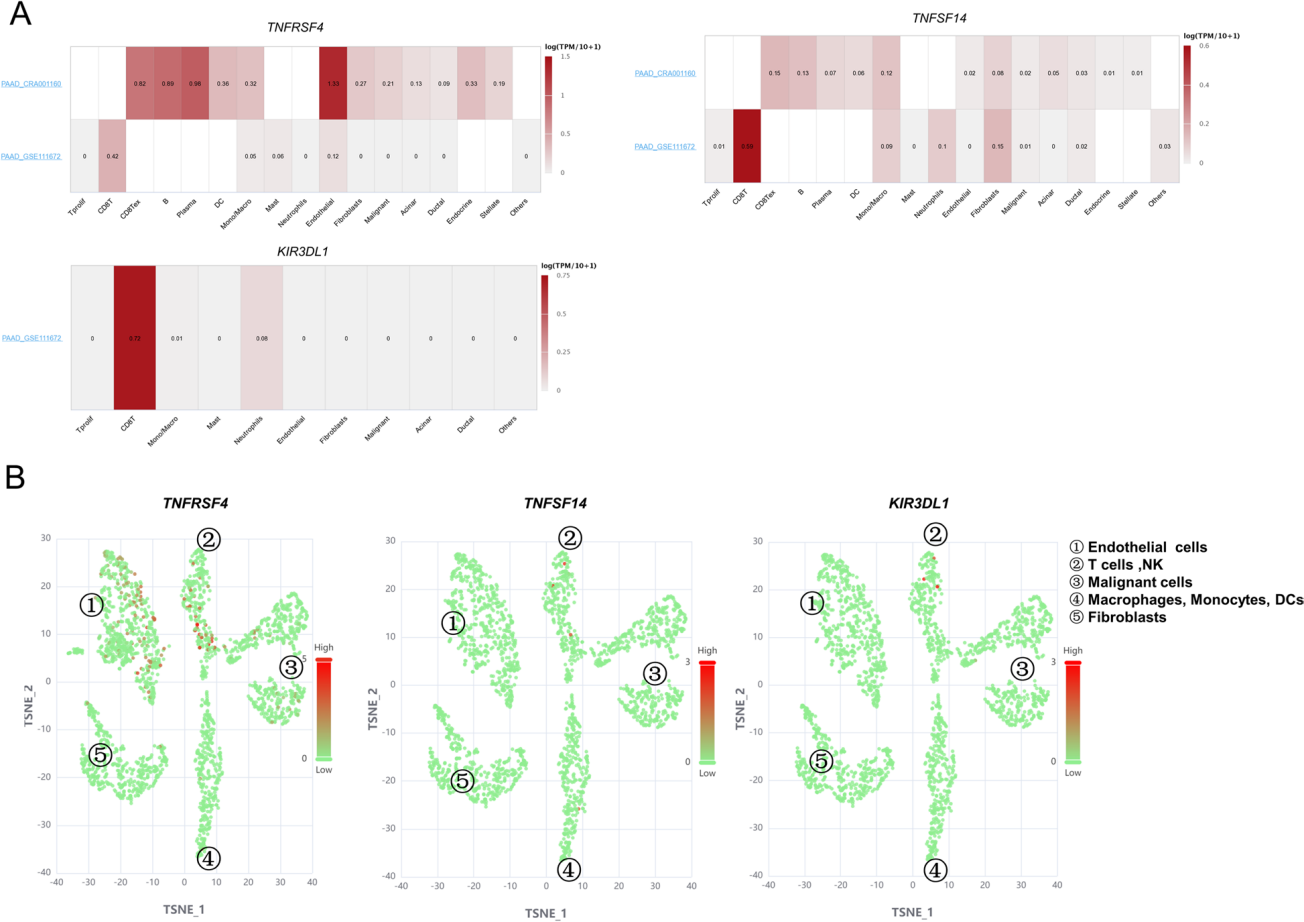
Single-cell RNA-Seq data of PAAD. **A** Single-cell RNA-Seq data of two PAAD datasets in TISCH database. **B** t-SNE of normalized single-cell RNA-seq data for three immune checkpoints in CancerSEA database

Single-cell RNA-seq data from two datasets (PAAD_CRA001160 and PAAD_GSE111672) of the TISCH database was used to evaluate this three-immune checkpoints (*TNFRSF4*, *TNFRSF14* and *KIR3DL1*) expression in PAAD. *TNFRSF4* was mainly expressed on CD8 T cells, B cells as well as endothelial cells in both datasets (Fig. 8A). *TNFRSF14* was mainly expressed on CD8 T cells, B cells and Monocytes/Macrophages. *KIR3DL1* was expressed on CD8 T cells in PAAD_GSE111672. We also visualized the single-cell expression of these genes by t-SNE after single-cell clustering in CancerSCEM database. According to the results provided in the CancerSCEM database, total cells belong to 5 main clusters in PDAC samples. The expression of *TNFRSF4*, *TNFRSF14* and *KIR3DL1* were colored according to their expression patterns in each cluster (Fig. 8B). We found the three genes had specifc expression characteristics in single-cell level, which is relatively low in malignant cells compared to expression level in immune cells.

### TNFRSF4/OX40 was an independent prognosis-related gene for PAAD

**Fig. 9:**
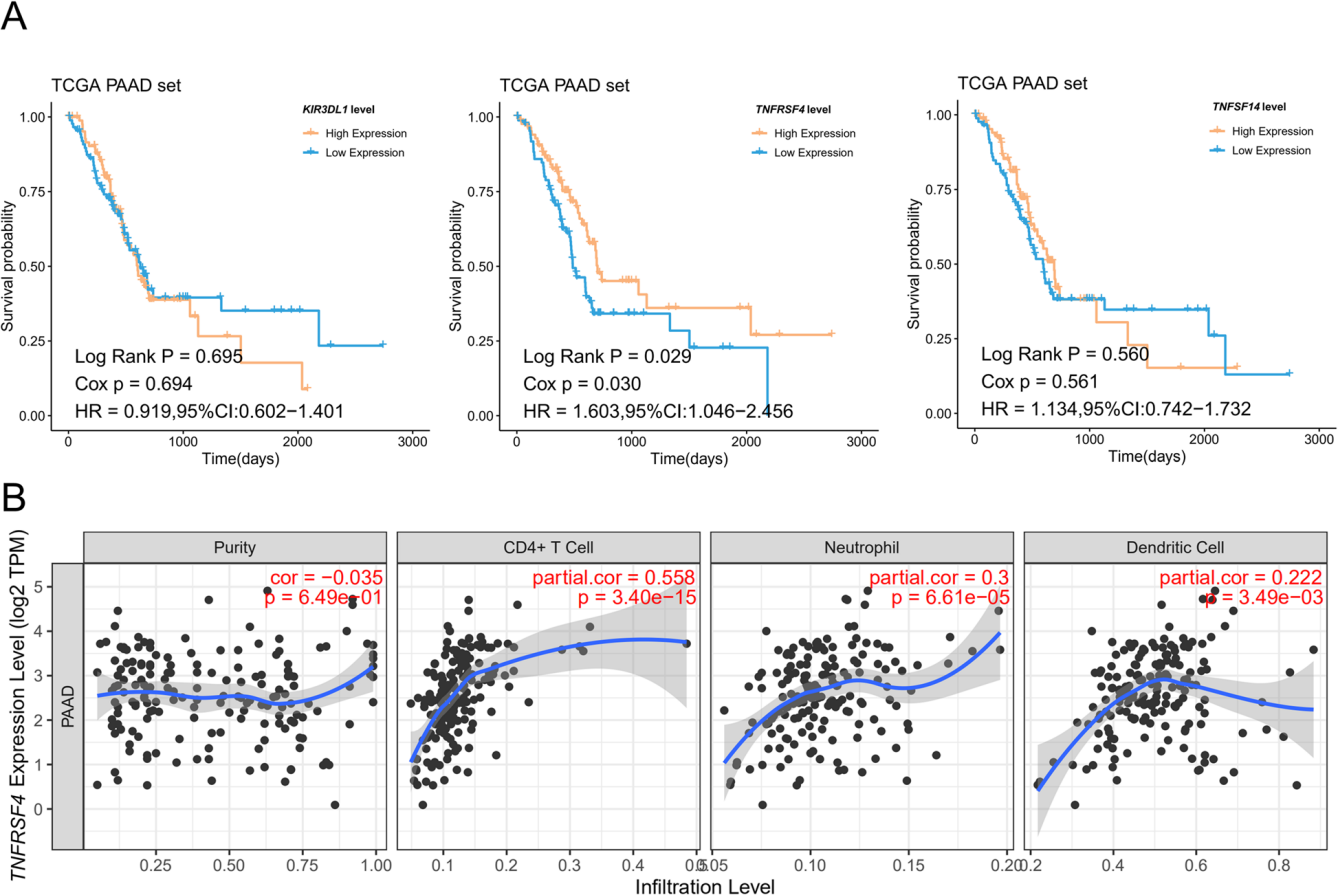
Survival and immune infiltration analysis of prognostic-related genes in PAAD. **A** Kaplan–Meier plot of overall survival between differentially expressed *TNFRSF4*, *KIR3DL1*, and *TNFRSF14* groups. **B** Correlation analysis between the expression level of *TNFRSF4* and immune infiltration

Further, to identify key genes related to the prognosis of PAAD patients, we analyzed the overall survival between differentially expressed immune checkpoint (*OX40*, *KIR3DL1*, and *TNFRSF14*) groups. Patients with higher *OX40* expression levels exhibited a significantly increased survival rate compared with those expressing less *OX40* (*p* < 0.05, Fig. 9A). No difference in overall survival was found in differentially expressed *KIR3DL1* (*p* = 0.695) and *TNFRSF14* groups (*p* = 0.56). According to the TIMER database analysis, *OX40* was correlated with CD4 + T cells, neutrophils, and dendritic cells infiltration, suggesting that *OX40* was also correlated with tumor immune microenvironment (Fig. 9B).

### Expression characteristics of OX40 in PAAD clinical specimens

**Fig. 10:**
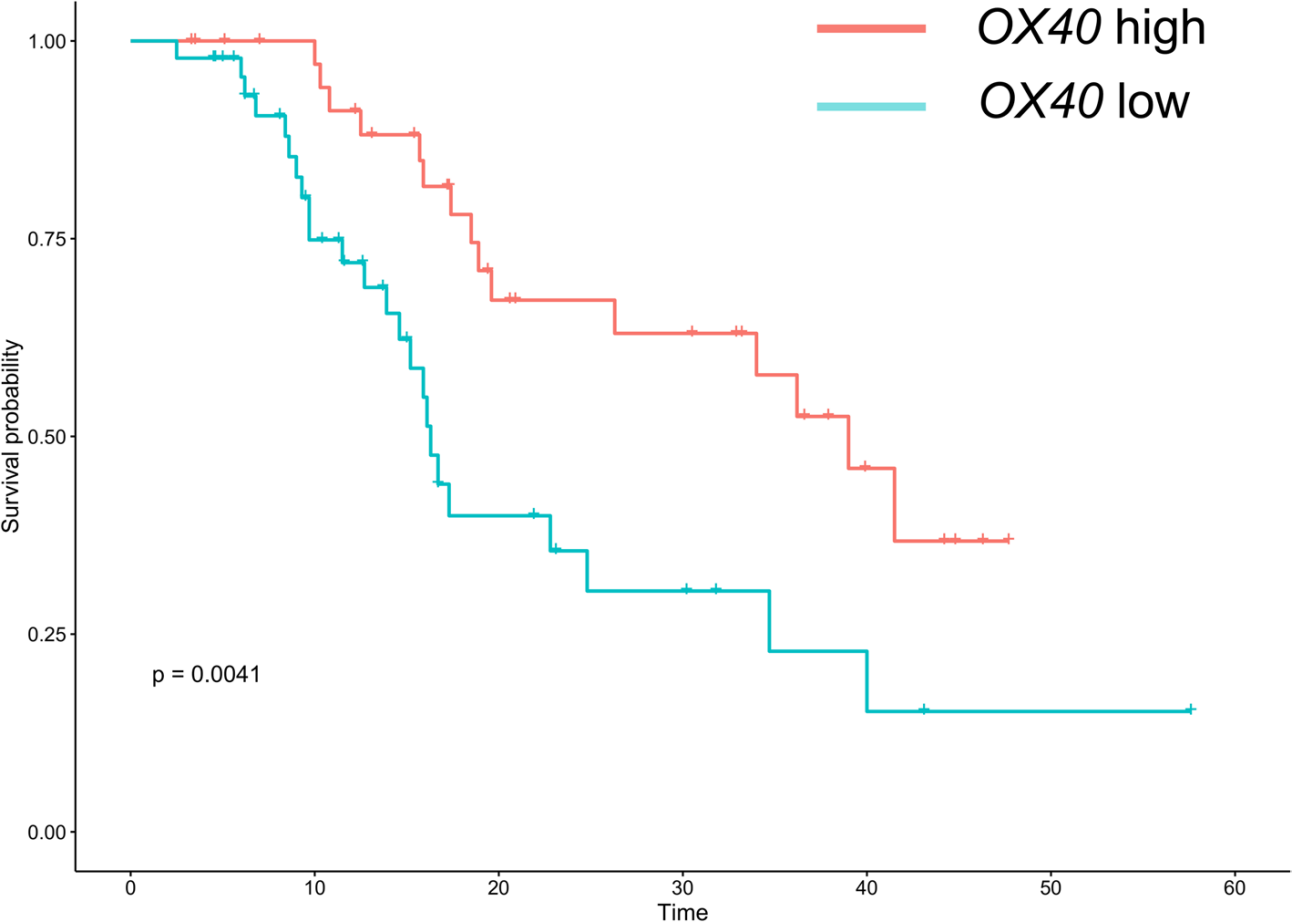
Survival analysis of *OX40* in clinical PAAD specimens. Kaplan–Meier plot between different *OX40* expressions in clinical PAAD specimens

**Fig. 11:**
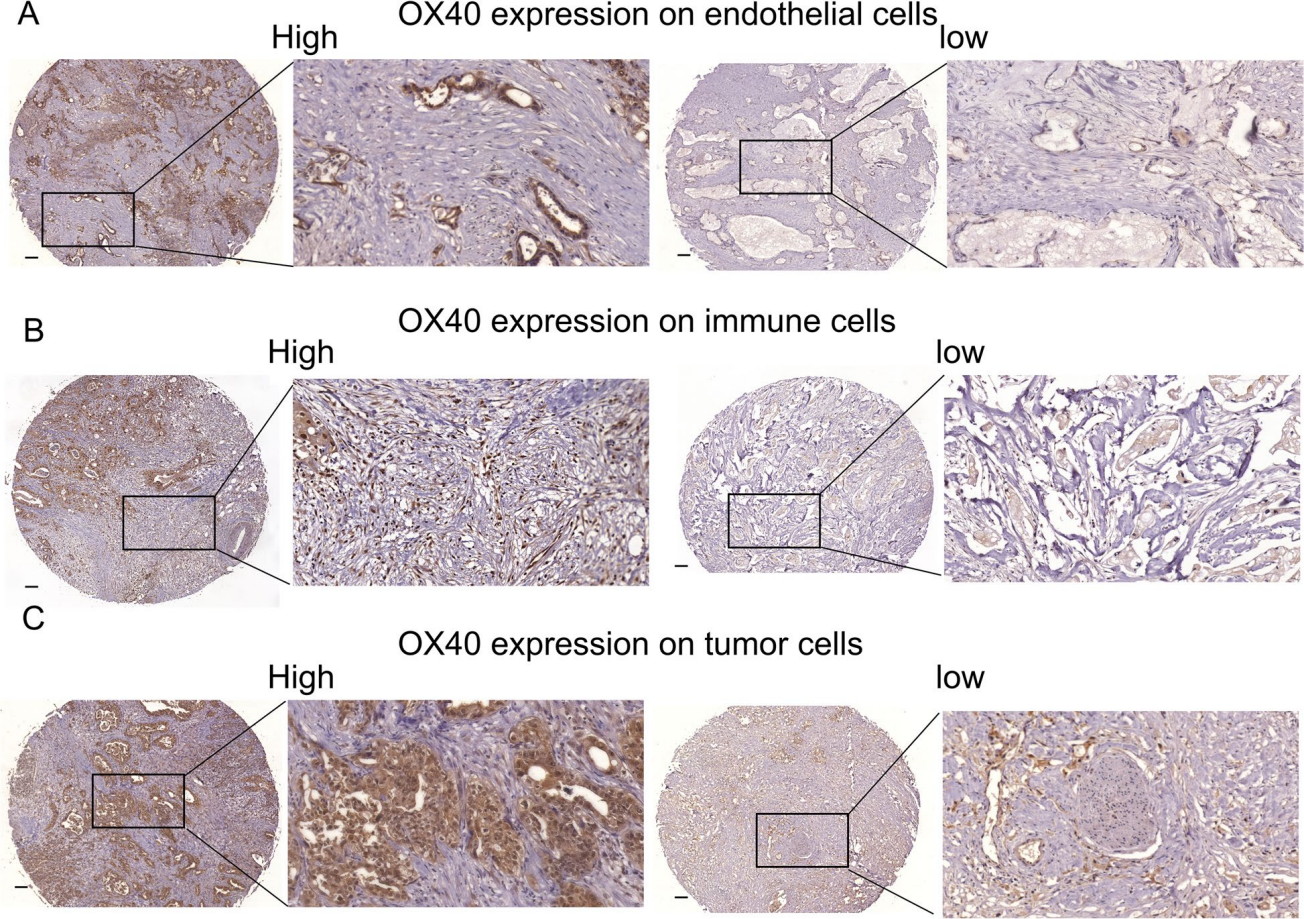
IHC staining of OX40 in clinical PAAD specimens. Human PAAD tissue slides were stained with anti-OX40 antibody. Magnifcation (10 × and 40x) images of high and low expression of OX40 were showed. Scale bar = 100 μm (black line at the bottom left). **A** OX40 expression on endothelial cells. **B** OX40 expression on immune cells. **C** OX40 expression on tumor cells

We tested the expression level of *OX40* in our 84 clinical specimens from PAAD tissues. Higher mRNA expression levels of *OX40* were associated with better survival in PAAD patients (*p* < 0.0041, Fig. 10). Through IHC, the protein expression of OX40 was found on TIICs, endothelial cells and tumor cells (Fig. 11), which is consistent with the data from single-cell RNA-seq data.

## Discussion

PAAD is one of the most aggressive cancers with a mortality rate almost equal to its morbidity. Moreover, the PAAD incidence and mortality are rising in many countries, inducing a substantial global burden. However, its treatment remains challenging. Currently, only limited treatment options are available for PAAD patients, providing modest improvements in their prognosis [12]. At present, a trustworthy molecular marker with a high prognostic value has not yet been determined for PAAD, and clinical staging and histopathological classification are the main criteria for prognosis. However, patients with similar clinical features have a different prognosis, which implies that these traditional methods are not enough for precise prognosis predictions. Therefore, the purpose of this study was to investigate the significance of immune checkpoint-related genes in PAAD prognosis and identify their unique prognostic significance in PAAD patients.

It has been reported that immune-related genes constitute robust prognostic indicators. The tumor immune microenvironment indicates the effects of immunotherapy and is closely related to patients’ prognoses [13]. Additionally, previous studies have evaluated the value of genetic signatures based on immune-related genes for malignancy tumors [14–16]. For example, our previous study regarding colon cancer presented a risk score model derived from four immune cells and was an independent predictive factor [11]. Yi et al. constructed a prognostic immune signature for lung adenocarcinoma comprehending 17-immune-related genes and validated its predictive capability [17]. Moreover, immune checkpoints-based scoring systems can be used to predict prognosis and select adjuvant therapies for tumor patients. Zelin Hou et al. reported a high expression level of the novel immune checkpoint protein- V-domain Ig suppressor of T cell activation (VISTA) in tumor-infiltrating CD68 + macrophages in PAAD [18]. In this study, VISTA was associated with a favorable PAAD prognosis. However, since immunosuppression in the tumor environment is not only affected by a single factor, the accuracy of experiments based on only one molecule is not satisfactory. The complexity of tumor biology and the immune environment of PAAD make it unlikely to define a single biomarker for prognosis [19]. A previous study integrated multiple immune checkpoints to establish a comprehensive immune scoring system that can help to improve prognosis prediction accuracy in gastric cancer [20]. Therefore, it was meaningful to investigate a prognostic evaluation model for PAAD patients based on multiple immune checkpoints.

PAAD has been labeled as nonimmunogenic cancer due to its unique tumor microenvironment, consisting of a dense fibrotic stroma and a scarcity of tumor-infiltrating lymphocytes [21, 22]. Therefore, despite the promising results of novel immunotherapies in multiple solid tumors, trials with single-agent immunotherapies in PAAD have been disappointing [23]. Moreover, several cell types such as macrophages, myeloid-derived suppressor cells (MDSC), T regulatory cells (Tregs) as well as fibroblasts have been reported to contribute to the immunosuppressive pancreatic cancer microenvironment [23]. In the present study, PAAD tissues demonstrated only a modest difference in the numbers of TIICs compared with normal tissues. This further demonstrated the highly tolerant and “immune quiescent” tumor microenvironment of PAAD.

Hence, a detailed and comprehensive evaluation of immune checkpoint gene expression in PAAD was performed on data derived from the TCGA database. In our current study, the expression of 12 immune checkpoint genes (*CD40*, *TNFRSF4*, *CD86*, *LAIR1*, *TNFRSF14*, *HAVCR2*, *CD244*, *TMIGD2*, *TNFRSF9*, *KIR3DL1*, *TNFRSF8*, and *CD48*) were downregulated in PAAD tissues compared with the normal group. It has been reported that CD40 and PLAU are involved in pancreatic cancer pathogenesis. Also, these genes play an important role in prognosis prediction [24, 25]. It was reported that CD40 agonists can mediate tumor regression in pancreatic ductal adenocarcinoma in mice and patients [26], in which the underlying immune mechanism can be both T-cell-dependent and T-cell-independent. In the present study, *CD40* was downregulated in PAAD groups compared with normal tissues. Three immune checkpoint genes, *OX40* (*TNFRSF4*), *TNFSF14*, and *KIR3DL1*, were filtered using the LASSO method to build a three-gene immune-related risk score model with maximum prognostic value. Satisfyingly, in the validation dataset, this risk score model showed a good prediction value with AUC = 0.941 for 3-year OS and 0.865 for 5-year OS. The risk score for each patient was also calculated. The KM curves showed significant differences between high-risk and low-risk patients. The immune checkpoint-related risk scoring model demonstrated strong predicting ability in the training, testing, and total sets. This risk score model was also applied to a GEO dataset for validation. When clinicopathological factors (age, gender, grade, and TNM stages) were combined, both univariate and multivariate Cox analyses showed that this immune-related risk score acted as an independent factor for PAAD prognosis. Finally, a nomogram was established for clinical practice based on the multivariate Cox analysis coefficients.

We explored the expression pattern of three filtered genes in single-cell level in TISCH and CancerSCEM database. *TNFRSF4*, *TNFRSF14* and *KIR3DL1* had specifc expression characteristics which expression is relatively high in immune cells compared to malignant cells. Relatively higher *TNFRSF4* expression level was observed in CD8 T cells,endothelial cells, and B/plasma cells. *TNFSF14* and *KIR3DL1* were mainly expressed on CD8 T cells. T-SNE plot showed same trend of these three genes expression in the pancreatic cancer. It was reported *OX40* was expressed predominantly on activated CD4^+^ /CD8^+^ T cells, neutrophils, and NK cells [27]. In PAAD CRA001160 of TISCH and PDAC-027 of CancerSCEM, *OX40* expression was relatively high on endothelial cells, which might explain the heterogeneity in pancreatic cancer microenvironment.

Furthermore, this immune checkpoints-based risk score model presented links to TIICs in our manuscript. The CIBERSORT algorithm was used to estimate the relative abundance of 22 TIICs in each PAAD sample. Our results showed that immune infiltration levels of naive B cells, CD8 + T cells, and Tregs decrease in the high-risk group, which was associated with longer survival. Meanwhile, the infiltration levels of M2 macrophages, resting NK, and resting mast cells increased and were associated with poorer survival. Consistent with previous studies, the infiltration level of CD8 T cells was associated with better OS. Nonetheless, the role of Tregs and resting mast cells in PAAD prognosis remains unclear.

Functional enrichment analysis was performed with GSEA to identify the most significant functional items. The high-risk group was significantly associated with different immune pathways including glycolysis, IL-2-STAT5, IL-6-STAT3, and mTORC1 signaling [28]. Additionally, we found that the tumor mutation burden and ICD-related genes were markedly different between high and low-risk score groups. These results might partly explain the model’s predictive value.

In the present study, *OX40*, *TNFSF14*, and *KIR3DL1* were related to a good PAAD prognosis. The immune checkpoint OX40 acts as a key costimulatory molecule whose expression depends on complete T cell activation [29]. OX40 activation on T cells needs interaction with OX40L. Then, the OX40/OX40L signaling transmits a costimulatory signal and promotes T cells development, differentiation, and physiological functions. Recently, anti-OX40 was proved to enhance CD8 + T cells and reduce Treg infiltration in different tumors [30–32]. Moreover, *OX40* was postulated as an independent PAAD prognostic predictor in our study and related with infiltration of several immune cells. Then, we validated the prognostic ability of *OX40* in our PAAD clinical specimens. Higher *OX40* expression anticipates a favorable outcome in PAAD patients. Thus, OX40 can be a potential molecular target for PAAD treatments. Further experiments are needed to determine the function and mechanism of OX40 in PAAD.

Additionally, we identified *TNFSF14*, also known as *LIGHT*, in PAAD samples. The *TNFSF14* expression was largely found on immune cells such as activated T cells, NK, and immature DCs as well as several cancer cells [33]. Besides, tumor expression of TNFSF14 has a significant impact on host anti-tumor immune responses and contributes to tumor microenvironment remodeling [34]. TNFSF14 also helps establish anti-tumor memory by stimulating the function of effector and CD8^+^ T cells infiltration. It has been shown that targeting TNFSF14 signaling might enhance tumor lymphocyte infiltration [35]. A previous study showed that adipose-derived stem cells pre-loaded with TNFSF14-expressing myxoma virus mediate improved tumor regression in pancreatic ductal adenocarcinoma [36].

Killer cell immunoglobulin-like receptors (KIRs) are a family of transmembrane glycoproteins. The expression of KIRs was found on NK and T cells subpopulations. NK cell-mediated cytotoxicity is regulated by the inhibitory KIRs [37, 38] and a previous study found PAAD patients with elevated *KIR3DL1* expression on NK cells [39].

The limination of this study is that in the TCGA dataset PAAD contains several types of pancreatic cancer, of which PDAC accounts for the main part (163/196). This heterogeneity may cause some bias and should be avoided in the future study. Overall, the present study represents a novel strategy based on the combination of immune checkpoints to evaluate the prognosis of PAAD patients. The immune checkpoint score was able to distinguish PAAD patients with different prognoses. Also, this immune signature shed new light on PAAD molecular mechanisms and prognosis predictions. Finally, we identified *OX40* as a novel prognostic gene for PAAD, which can be integrated for precise prognosis prediction and risk stratification in the future.

## Conclusion

In the present study, we presented a risk score system based on three immune checkpoints (*OX40*/*TNFRSF4*, *TNFSF14*, and *KIR3DL1*) through LASSO and Cox regression analyses for prognostic prediction. The risk score model performed well regarding overall survival (OS) predictions. ScRNA-seq data showed the expression of the three genes in PAAD samples. Additionally, we identified *OX40* as an independent prognosis-related gene, and a higher OX40 expression was associated with increased survival rate and immune cells infiltration. Furthermore, we verified the prognosis ability of *OX40* in our clinical specimens. We also showed the protein expression of OX40 by immunohistochemistry (IHC) staining. Thus, our findings strongly suggested that the three-immune checkpoint score system as well as OX40 might be useful in the prognosis and design of personalized treatments for PAAD patients.

## Method

### Data collection

We identified and obtained gene expression data of the PAAD cohort from TCGA (https://portal.gdc.cancer.gov/), including 179 tumor cases and 4 normal cases. Correlative clinical data were also extracted. In addition, 167 cases normal pancreatic tissue were obtained from GETX database for analysis. According to CIBERSORT algorithm, samples with P value > 0.05 were screened out, and 123 pancreatic cancer samples were included in the analysis. Furthermore, GSE62452 dataset and ICGC database were used to verify our analysis results. In the end, the transcriptome data were analyzed with “limma” R package. We obtained the list of immune related genes from ImmPort database (https://www.immport.org/).

### Immune checkpoint-related risk scoring model construction

First, we randomly divided the total TCGA samples into a training cohort (63 patients) and a testing cohort (60 patients). The training cohort was used to seek prognostic immune checkpoint genes and develop the risk score model. Then, the testing and total cohorts were used to check the robustness and predictive power of the model. We evaluated independent prognostic risk factors using LASSO and Cox regression analyses. Then, we classified the patients into a high and a low-risk group by setting the median risk score as the cutoff value. The predictive power of the model and the genes identified were calculated by the Kaplan–Meier method. A Log-rank *p*-value < 0.05 was considered significant. To evaluate the prognostic value, we used ROC curves to assess the specificity and sensitivity of this recurrence risk model. Also, a comprehensive nomogram was established to predict prognosis using Cox analysis coefficients.

### Clinical specimens and IHC for OX40

A total of 84 PAAD samples were collected from January 2013 to April 2017 at the Department of Pancreatic Surgery, Fudan University Shanghai Cancer Center of China. All patients included had resectable PAAD without distant metastasis. Patients who received preoperative anticancer treatment were excluded. The time between the date of surgery to death of all causes or the last follow-up date was calculated as the overall survival (OS). All patients were followed to June 2018. Informed consent was obtained from all patients.The Clinical Research Ethics Committee of Shanghai Cancer Center of Fudan University approved this study.

Protein expression of OX40 in PAAD specimens was measured with IHC. Slides were routinely dewaxed and under antigen retrieval. Hydrogen peroxide was used to block endogenous peroxidase activity and fetal bovine serum for blocking. Primary antibody was used to detect OX40 (Invitrogen, 1 µg/mL). At last, diaminobenzidine colorimetry and digital microscope were applied to quantify OX40 expression levels.

### Pathway and gene set enrichment analysis (GSEA)

Gene ontology (GO) functional and Kyoto Encyclopedia of Genes and Genomes (KEGG) pathway enrichment analyses were performed with the DAVID database and its online analysis tool (https://david.ncifcrf.gov/). The GO and KEGG pathway terms were considered significantly with *p*-value < 0.05. The “Ggplot2” statistical plotting R package was used to generate visualizations.

### Assessment of TIICs

The CIBERSORT algorithm was used to estimate the relative abundance of 22 TIICs in each PAAD sample as previously reported (http://cibersort.stanford.edu). The corrected transcriptome data was uploaded to CIBERSORT and the algorithm was configured with 1000 rows. A *p-*value < 0.05 was considered statistically significant.

### Mutation analysis

We retrieved genetic mutation data of PAAD patients from the TCGA database. The somatic mutation data were analyzed and summarized using maftools*.*

### Single-cell mRNA-sequencing data analysis

We used two online scRNA-seq database (TISCH and *Cance*rSEA) to explore the key immune checkpoints genes expression of risk score model in single-cell level. Tumor Immune Single-cell Hub (TISCH) is a scRNA-seq database focusing on tumor microenvironment [40] (http://tisch.comp-genomics.org/home/). TISCH collected data from GEO and ArrayExpress. It involves of 2,045,746 cancer single cells in 28 cancer types. After selecting a dataset, a heatmap and a violin plot can be displayed to reflect the gene expression (logTPM) in different cell types. In this manuscript, we used two PAAD datasets (CRA001160 and GSE111672) in TISCH to decipher the different expression patterns of three immune checkpoints in PAAD at single cell level. CancerSEA is a cancer database for investigating tumor microenvironment at single-cell transcriptome level [41] (http://biocc.hrbmu.edu.cn/CancerSEA/home.jsp). The database contains 20 cancer types covering 208 human samples (28 projects). In this manuscript, the unsupervised cell clustering in tSNE manner were analyzed in PAAD sample(project ID PDAC-027). Then *TNFRSF4*, *TNFSF14* and *KIR3DL1* expression were presented using cancer single cell data.

### RNA isolation and quantitative real-time PCR

Total RNA was isolated from frozen pancreatic tissues using TRIzol and converted to cDNA using a PrimeScript RT Master Mix as previously described [42, 43]. Quantitative real-time PCR for *OX40* was performed using SYBR green (Applied Biosystems). The melting curve analysis was used to confirm the specificity of real-time PCR.

### Statistical analyses

Statistical analyses in the manuscipt were performed with R (version 3.6.1). Independent Student’s t-tests were used to compare continuous variables with normal distribution between groups, while the Mann–Whitney U-test was used to compare variables with skewed distribution. The Pearson correlation coefficient was used in the correlation matrix. A two-sided *p*-value < 0.05 was considered statistically significant.

## Supplementary Information

**Additional file 1**.

## Data Availability

The gene expression data of PAAD cohort are derived from publicly available databases TCGA (https://portal.gdc.cancer.gov/). Single-cell mRNA-sequencing data were from two online scRNA-seq database TISCH (http://tisch.comp-genomics.org/home/)and*Cance*rSEA (http://biocc.hrbmu.edu.cn/CancerSEA/home.jsp).
