# Supplementary material for: A novel immune checkpoint score system for prognostic evaluation in pancreatic adenocarcinoma
Source: BMC Gastroenterol. 2023 Apr 6;23:113. doi: 10.1186/s12876-023-02748-w (PMC10080823; doi:10.1186/s12876-023-02748-w)
Supplement: Supplementary file 1 — Additional file 1. [file 12876_2023_2748_MOESM1_ESM.pdf]

sample	cancer type abbreviation	histological_type
TCGA-2J-AAB1-01	PAAD	Pancreas-Adenocarcinoma-Other Subtype
TCGA-2J-AAB4-01	PAAD	Pancreas-Adenocarcinoma-Other Subtype
TCGA-2J-AAB6-01	PAAD	Pancreas-Adenocarcinoma Ductal Type
TCGA-2J-AAB8-01	PAAD	Pancreas-Adenocarcinoma Ductal Type
TCGA-2J-AAB9-01	PAAD	Pancreas-Adenocarcinoma Ductal Type
TCGA-2J-AABA-01	PAAD	Pancreas-Adenocarcinoma Ductal Type
TCGA-2J-AABE-01	PAAD	Pancreas-Adenocarcinoma Ductal Type
TCGA-2J-AABF-01	PAAD	Pancreas-Adenocarcinoma Ductal Type
TCGA-2J-AABH-01	PAAD	Pancreas-Adenocarcinoma-Other Subtype
TCGA-2J-AABI-01	PAAD	Pancreas-Adenocarcinoma-Other Subtype
TCGA-2J-AABK-01	PAAD	Pancreas-Adenocarcinoma Ductal Type
TCGA-2J-AABO-01	PAAD	Pancreas-Adenocarcinoma Ductal Type
TCGA-2J-AABP-01	PAAD	Pancreas-Undifferentiated Carcinoma
TCGA-2J-AABR-01	PAAD	Pancreas-Adenocarcinoma Ductal Type
TCGA-2J-AABT-01	PAAD	Pancreas-Adenocarcinoma Ductal Type
TCGA-2J-AABU-01	PAAD	Pancreas-Adenocarcinoma Ductal Type
TCGA-2J-AABV-01	PAAD	Pancreas-Adenocarcinoma Ductal Type
TCGA-2L-AAQA-01	PAAD	Pancreas-Adenocarcinoma Ductal Type
TCGA-2L-AAQE-01	PAAD	Pancreas-Adenocarcinoma Ductal Type
TCGA-2L-AAQI-01	PAAD	Pancreas-Adenocarcinoma Ductal Type
TCGA-2L-AAQJ-01	PAAD	Pancreas-Adenocarcinoma Ductal Type
TCGA-2L-AAQL-01	PAAD	Pancreas-Adenocarcinoma Ductal Type
TCGA-2L-AAQM-01	PAAD	Pancreas-Adenocarcinoma-Other Subtype
TCGA-3A-A9I5-01	PAAD	Pancreas-Adenocarcinoma Ductal Type
TCGA-3A-A9I7-01	PAAD	Pancreas-Adenocarcinoma Ductal Type
TCGA-3A-A9I9-01	PAAD	Pancreas-Adenocarcinoma Ductal Type
TCGA-3A-A9IB-01	PAAD	Pancreas-Adenocarcinoma Ductal Type
TCGA-3A-A9IC-01	PAAD	Pancreas-Adenocarcinoma Ductal Type
TCGA-3A-A9IH-01	PAAD	Pancreas-Adenocarcinoma Ductal Type
TCGA-3A-A9IJ-01	PAAD	Pancreas-Adenocarcinoma-Other Subtype
TCGA-3A-A9IL-01	PAAD	Pancreas-Adenocarcinoma-Other Subtype
TCGA-3A-A9IN-01	PAAD	Pancreas-Adenocarcinoma-Other Subtype
TCGA-3A-A9IO-01	PAAD	Pancreas-Adenocarcinoma-Other Subtype
TCGA-3A-A9IR-01	PAAD	Pancreas-Adenocarcinoma-Other Subtype
TCGA-3A-A9IS-01	PAAD	Pancreas-Adenocarcinoma-Other Subtype
TCGA-3A-A9IU-01	PAAD	Pancreas-Adenocarcinoma Ductal Type
TCGA-3A-A9IV-01	PAAD	Pancreas-Adenocarcinoma-Other Subtype
TCGA-3A-A9IX-01	PAAD	Pancreas-Adenocarcinoma Ductal Type
TCGA-3A-A9IZ-01	PAAD	Pancreas-Adenocarcinoma Ductal Type
TCGA-3A-A9J0-01	PAAD	Pancreas-Adenocarcinoma Ductal Type
TCGA-3E-AAAY-01	PAAD	Pancreas-Adenocarcinoma Ductal Type
TCGA-3E-AAAZ-01	PAAD	Pancreas-Adenocarcinoma Ductal Type
TCGA-F2-6879-01	PAAD	Pancreas-Adenocarcinoma-Other Subtype
TCGA-F2-6880-01	PAAD	Pancreas-Adenocarcinoma Ductal Type
TCGA-F2-7273-01	PAAD	Pancreas-Adenocarcinoma Ductal Type
TCGA-F2-7276-01	PAAD	Pancreas-Adenocarcinoma Ductal Type
TCGA-F2-A44G-01	PAAD	Pancreas-Adenocarcinoma Ductal Type
TCGA-F2-A44H-01	PAAD	Pancreas-Adenocarcinoma Ductal Type
TCGA-F2-A7TX-01	PAAD	Pancreas-Adenocarcinoma Ductal Type
TCGA-F2-A8YN-01	PAAD	Pancreas-Adenocarcinoma Ductal Type
TCGA-FB-A4P5-01	PAAD	Pancreas-Adenocarcinoma Ductal Type
TCGA-FB-A4P6-01	PAAD	Pancreas-Adenocarcinoma Ductal Type
TCGA-FB-A545-01	PAAD	Pancreas-Adenocarcinoma Ductal Type
TCGA-FB-A5VM-01	PAAD	Pancreas-Adenocarcinoma Ductal Type
TCGA-FB-A78T-01	PAAD	Pancreas-Adenocarcinoma Ductal Type
TCGA-FB-A7DR-01	PAAD	Pancreas-Colloid (mucinous non-cystic) Carcinoma
TCGA-FB-AAPP-01	PAAD	Pancreas-Adenocarcinoma Ductal Type

TCGA-FB-AAPQ-01	PAAD	Pancreas-Adenocarcinoma Ductal Type
TCGA-FB-AAPS-01	PAAD	Pancreas-Adenocarcinoma Ductal Type
TCGA-FB-AAPU-01	PAAD	Pancreas-Adenocarcinoma Ductal Type
TCGA-FB-AAPY-01	PAAD	Pancreas-Adenocarcinoma Ductal Type
TCGA-FB-AAPZ-01	PAAD	Pancreas-Adenocarcinoma Ductal Type
TCGA-FB-AAQ0-01	PAAD	Pancreas-Adenocarcinoma Ductal Type
TCGA-FB-AAQ1-01	PAAD	Pancreas-Adenocarcinoma Ductal Type
TCGA-FB-AAQ2-01	PAAD	Pancreas-Adenocarcinoma Ductal Type
TCGA-FB-AAQ3-01	PAAD	Pancreas-Adenocarcinoma Ductal Type
TCGA-FB-AAQ6-01	PAAD	Pancreas-Adenocarcinoma Ductal Type
TCGA-FZ-5919-01	PAAD	Pancreas-Adenocarcinoma Ductal Type
TCGA-FZ-5919-11	PAAD	Pancreas-Adenocarcinoma Ductal Type
TCGA-FZ-5920-01	PAAD	Pancreas-Adenocarcinoma Ductal Type
TCGA-FZ-5920-11	PAAD	Pancreas-Adenocarcinoma Ductal Type
TCGA-FZ-5921-01	PAAD	Pancreas-Adenocarcinoma Ductal Type
TCGA-FZ-5922-01	PAAD	Pancreas-Adenocarcinoma Ductal Type
TCGA-FZ-5922-11	PAAD	Pancreas-Adenocarcinoma Ductal Type
TCGA-FZ-5923-01	PAAD	Pancreas-Adenocarcinoma Ductal Type
TCGA-FZ-5923-11	PAAD	Pancreas-Adenocarcinoma Ductal Type
TCGA-FZ-5924-01	PAAD	Pancreas-Adenocarcinoma Ductal Type
TCGA-FZ-5924-11	PAAD	Pancreas-Adenocarcinoma Ductal Type
TCGA-FZ-5926-01	PAAD	Pancreas-Adenocarcinoma Ductal Type
TCGA-FZ-5926-11	PAAD	Pancreas-Adenocarcinoma Ductal Type
TCGA-H6-8124-01	PAAD	Pancreas-Adenocarcinoma Ductal Type
TCGA-H6-8124-11	PAAD	Pancreas-Adenocarcinoma Ductal Type
TCGA-H6-A45N-01	PAAD	Pancreas-Adenocarcinoma-Other Subtype
TCGA-H6-A45N-11	PAAD	Pancreas-Adenocarcinoma-Other Subtype
TCGA-H8-A6C1-01	PAAD	Pancreas-Adenocarcinoma-Other Subtype
TCGA-HV-A5A3-01	PAAD	Pancreas-Adenocarcinoma Ductal Type
TCGA-HV-A5A3-11	PAAD	Pancreas-Adenocarcinoma Ductal Type
TCGA-HV-A5A4-01	PAAD	Pancreas-Adenocarcinoma Ductal Type
TCGA-HV-A5A5-01	PAAD	Pancreas-Adenocarcinoma Ductal Type
TCGA-HV-A5A6-01	PAAD	Pancreas-Adenocarcinoma Ductal Type
TCGA-HV-A7OL-01	PAAD	Pancreas-Adenocarcinoma Ductal Type
TCGA-HV-A7OP-01	PAAD	Pancreas-Adenocarcinoma-Other Subtype
TCGA-HV-AA8V-01	PAAD	Pancreas-Adenocarcinoma Ductal Type
TCGA-HV-AA8X-01	PAAD	Pancreas-Adenocarcinoma Ductal Type
TCGA-HZ-7289-01	PAAD	Pancreas-Adenocarcinoma-Other Subtype
TCGA-HZ-7918-01	PAAD	Pancreas-Adenocarcinoma Ductal Type
TCGA-HZ-7919-01	PAAD	Pancreas-Adenocarcinoma Ductal Type
TCGA-HZ-7920-01	PAAD	Pancreas-Adenocarcinoma Ductal Type
TCGA-HZ-7922-01	PAAD	Pancreas-Adenocarcinoma Ductal Type
TCGA-HZ-7923-01	PAAD	Pancreas-Adenocarcinoma Ductal Type
TCGA-HZ-7924-01	PAAD	Pancreas-Adenocarcinoma Ductal Type
TCGA-HZ-7925-01	PAAD	Pancreas-Adenocarcinoma Ductal Type
TCGA-HZ-7926-01	PAAD	Pancreas-Adenocarcinoma Ductal Type
TCGA-HZ-8001-01	PAAD	Pancreas-Adenocarcinoma Ductal Type
TCGA-HZ-8002-01	PAAD	Pancreas-Adenocarcinoma Ductal Type
TCGA-HZ-8003-01	PAAD	Pancreas-Adenocarcinoma Ductal Type
TCGA-HZ-8005-01	PAAD	Pancreas-Adenocarcinoma Ductal Type
TCGA-HZ-8315-01	PAAD	Pancreas-Adenocarcinoma Ductal Type
TCGA-HZ-8317-01	PAAD	Pancreas-Adenocarcinoma Ductal Type
TCGA-HZ-8519-01	PAAD	Pancreas-Adenocarcinoma-Other Subtype
TCGA-HZ-8636-01	PAAD	Pancreas-Adenocarcinoma Ductal Type
TCGA-HZ-8637-01	PAAD	Pancreas-Adenocarcinoma Ductal Type
TCGA-HZ-8638-01	PAAD	Pancreas-Colloid (mucinous non-cystic) Carcinoma
TCGA-HZ-A49G-01	PAAD	Pancreas-Adenocarcinoma Ductal Type
TCGA-HZ-A49H-01	PAAD	Pancreas-Adenocarcinoma Ductal Type

TCGA-HZ-A49I-01	PAAD	Pancreas-Adenocarcinoma Ductal Type
TCGA-HZ-A4BH-01	PAAD	Pancreas-Adenocarcinoma Ductal Type
TCGA-HZ-A4BK-01	PAAD	Pancreas-Adenocarcinoma-Other Subtype
TCGA-HZ-A77O-01	PAAD	Pancreas-Adenocarcinoma Ductal Type
TCGA-HZ-A77P-01	PAAD	Pancreas-Adenocarcinoma-Other Subtype
TCGA-HZ-A77Q-01	PAAD	Pancreas-Adenocarcinoma-Other Subtype
TCGA-HZ-A8P0-01	PAAD	Pancreas-Adenocarcinoma Ductal Type
TCGA-HZ-A8P1-01	PAAD	Pancreas-Adenocarcinoma Ductal Type
TCGA-HZ-A9TJ-06	PAAD	Pancreas-Adenocarcinoma-Other Subtype
TCGA-HZ-A9TJ-01	PAAD	Pancreas-Adenocarcinoma-Other Subtype
TCGA-IB-7644-01	PAAD	Pancreas-Adenocarcinoma Ductal Type
TCGA-IB-7645-01	PAAD	Pancreas-Adenocarcinoma Ductal Type
TCGA-IB-7646-01	PAAD	Pancreas-Adenocarcinoma Ductal Type
TCGA-IB-7647-01	PAAD	Pancreas-Adenocarcinoma Ductal Type
TCGA-IB-7649-01	PAAD	Pancreas-Adenocarcinoma Ductal Type
TCGA-IB-7651-01	PAAD	Pancreas-Adenocarcinoma Ductal Type
TCGA-IB-7652-01	PAAD	Pancreas-Adenocarcinoma Ductal Type
TCGA-IB-7654-01	PAAD	Pancreas-Adenocarcinoma Ductal Type
TCGA-IB-7885-01	PAAD	Pancreas-Adenocarcinoma Ductal Type
TCGA-IB-7886-01	PAAD	Pancreas-Adenocarcinoma Ductal Type
TCGA-IB-7887-01	PAAD	Pancreas-Adenocarcinoma Ductal Type
TCGA-IB-7888-01	PAAD	Pancreas-Adenocarcinoma Ductal Type
TCGA-IB-7889-01	PAAD	Pancreas-Adenocarcinoma Ductal Type
TCGA-IB-7890-01	PAAD	Pancreas-Adenocarcinoma Ductal Type
TCGA-IB-7891-01	PAAD	Pancreas-Adenocarcinoma Ductal Type
TCGA-IB-7893-01	PAAD	Pancreas-Adenocarcinoma Ductal Type
TCGA-IB-7897-01	PAAD	Pancreas-Adenocarcinoma Ductal Type
TCGA-IB-8126-01	PAAD	Pancreas-Adenocarcinoma Ductal Type
TCGA-IB-8127-01	PAAD	Pancreas-Adenocarcinoma Ductal Type
TCGA-IB-A5SO-01	PAAD	Pancreas-Adenocarcinoma Ductal Type
TCGA-IB-A5SP-01	PAAD	Pancreas-Adenocarcinoma Ductal Type
TCGA-IB-A5SQ-01	PAAD	Pancreas-Adenocarcinoma Ductal Type
TCGA-IB-A5SS-01	PAAD	Pancreas-Adenocarcinoma Ductal Type
TCGA-IB-A5ST-01	PAAD	Pancreas-Adenocarcinoma Ductal Type
TCGA-IB-A6UF-01	PAAD	Pancreas-Adenocarcinoma Ductal Type
TCGA-IB-A6UG-01	PAAD	Pancreas-Adenocarcinoma Ductal Type
TCGA-IB-A7LX-01	PAAD	Pancreas-Adenocarcinoma Ductal Type
TCGA-IB-A7M4-01	PAAD	Pancreas-Adenocarcinoma Ductal Type
TCGA-IB-AAUM-01	PAAD	Pancreas-Adenocarcinoma Ductal Type
TCGA-IB-AAUN-01	PAAD	Pancreas-Adenocarcinoma Ductal Type
TCGA-IB-AAUO-01	PAAD	Pancreas-Adenocarcinoma Ductal Type
TCGA-IB-AAUP-01	PAAD	Pancreas-Adenocarcinoma Ductal Type
TCGA-IB-AAUQ-01	PAAD	Pancreas-Adenocarcinoma Ductal Type
TCGA-IB-AAUR-01	PAAD	Pancreas-Adenocarcinoma Ductal Type
TCGA-IB-AAUS-01	PAAD	Pancreas-Adenocarcinoma Ductal Type
TCGA-IB-AAUT-01	PAAD	Pancreas-Colloid (mucinous non-cystic) Carcinoma
TCGA-IB-AAUU-01	PAAD	Pancreas-Adenocarcinoma Ductal Type
TCGA-IB-AAUV-01	PAAD	Pancreas-Adenocarcinoma Ductal Type
TCGA-IB-AAUW-01	PAAD	Pancreas-Adenocarcinoma Ductal Type
TCGA-L1-A7W4-01	PAAD	Pancreas-Adenocarcinoma Ductal Type
TCGA-LB-A7SX-01	PAAD	Pancreas-Adenocarcinoma Ductal Type
TCGA-LB-A8F3-01	PAAD	Pancreas-Adenocarcinoma Ductal Type
TCGA-LB-A9Q5-01	PAAD	Pancreas-Adenocarcinoma Ductal Type
TCGA-M8-A5N4-01	PAAD	Pancreas-Adenocarcinoma Ductal Type
TCGA-OE-A75W-01	PAAD	Pancreas-Adenocarcinoma Ductal Type
TCGA-PZ-A5RE-01	PAAD	Pancreas-Adenocarcinoma Ductal Type
TCGA-Q3-A5QY-01	PAAD	Pancreas-Adenocarcinoma Ductal Type
TCGA-Q3-AA2A-01	PAAD	Pancreas-Adenocarcinoma Ductal Type

TCGA-RB-A7B8-01	PAAD	Pancreas-Adenocarcinoma-Other Subtype
TCGA-RB-AA9M-01	PAAD	Pancreas-Adenocarcinoma Ductal Type
TCGA-RL-AAAS-01	PAAD	Pancreas-Adenocarcinoma Ductal Type
TCGA-S4-A8RM-01	PAAD	Pancreas-Adenocarcinoma Ductal Type
TCGA-S4-A8RO-01	PAAD	Pancreas-Adenocarcinoma Ductal Type
TCGA-S4-A8RP-01	PAAD	Pancreas-Adenocarcinoma Ductal Type
TCGA-US-A774-01	PAAD	Pancreas-Adenocarcinoma Ductal Type
TCGA-US-A776-01	PAAD	Pancreas-Colloid (mucinous non-cystic) Carcinoma
TCGA-US-A779-01	PAAD	Pancreas-Adenocarcinoma Ductal Type
TCGA-US-A77E-01	PAAD	Pancreas-Adenocarcinoma-Other Subtype
TCGA-US-A77G-01	PAAD	Pancreas-Adenocarcinoma Ductal Type
TCGA-US-A77J-01	PAAD	Pancreas-Adenocarcinoma Ductal Type
TCGA-XD-AAUG-01	PAAD	Pancreas-Adenocarcinoma Ductal Type
TCGA-XD-AAUH-01	PAAD	Pancreas-Adenocarcinoma Ductal Type
TCGA-XD-AAUI-01	PAAD	Pancreas-Adenocarcinoma Ductal Type
TCGA-XD-AAUL-01	PAAD	Pancreas-Adenocarcinoma Ductal Type
TCGA-XN-A8T3-01	PAAD	Pancreas-Adenocarcinoma Ductal Type
TCGA-XN-A8T5-01	PAAD	Pancreas-Adenocarcinoma-Other Subtype
TCGA-YB-A89D-01	PAAD	Pancreas-Adenocarcinoma Ductal Type
TCGA-YB-A89D-11	PAAD	Pancreas-Adenocarcinoma Ductal Type
TCGA-YH-A8SY-01	PAAD	[Discrepancy]
TCGA-YY-A8LH-01	PAAD	Pancreas-Adenocarcinoma Ductal Type
TCGA-Z5-AAPL-01	PAAD	Pancreas-Adenocarcinoma Ductal Type
